# The Effects of Three Phenolic Substances on the Growth and Digestive Physiology of the Fall Armyworm *Spodoptera frugiperda* (Lepidoptera: Noctuidae)

**DOI:** 10.3390/insects16070669

**Published:** 2025-06-26

**Authors:** Jin-Yan Lv, Ya-Nan Deng, Xiao-Rong Liu, Dan Niu, Wan-Shu Zhang

**Affiliations:** 1 Dazhou Key Laboratory of Agricultural Resources Development and Ecological Conservation in Daba Mountain, Sichuan University of Arts and Science, Dazhou 635000, China; 2 College of Forestry, Northeast Forestry University, Harbin 150040, China

**Keywords:** vanillic acid, sinapic acid, syringic acid, *Spodoptera frugiperda*, antifeedants, ecofriendly prevention and control

## Abstract

The spread of invasive pests can exacerbate the threat of pest stress to agricultural production. Screening natural antifeedants from the secondary metabolites of plants may provide efficient alternative control methods for the integrated management of invasive pests. Therefore, in this study, the three phenolic substances were evenly mixed with the artificial diet at mass ratios of 1:200 and 1:40, respectively. The treatment groups were labeled as follows: vanillic acid (Va1: 1:200, Va2: 1:40), sinapic acid (Si1: 1:200, Si2: 1:40), and syringic acid (Sy1: 1:200, Sy2: 1:40). Subsequently, the feed intake, body weight, protein content, and digestive enzyme activity of the *S. frugiperda* feeding were measured. This research was extended to the pupal and adult stages, focusing on pupation rate, pupal weight, adult emergence rate, and adult longevity. The results showed that the food intake and body weight of the 4th–6th instars of *S. frugiperda* decreased significantly after feeding on the different ratios of phenolic substances. Although the protein accumulation of the 4th–5th-instar larvae was inhibited, the protein content of the 6th-instar larvae showed significant accumulation, with that of the Va2 and Sy1 treatment groups being 124.84% and 165.07% of that of the control, respectively. At the same time, the trypsin activity of the 4th–6th-instar larvae in different treatment groups significantly increased, while the activities of pepsin and α-amylase fluctuated but overall showed an increasing trend. The interference of vanillic acid, sinapic acid, and syringic acid on the digestion and absorption of the larvae limited the pupation rate but did not have a significant effect on the pupal weight. All three phenolic substances reduced the adult emergence rate, but unlike the sinapic acid and syringic acid treatments, vanillic acid also had a negative impact on adult longevity. These results can provide material reserves for the development of antifeedants for *S. frugiperda*, and can also provide optimal plans for its ecofriendly prevention and control.

## Simple Summary

Previous studies have shown that vanillic acid, sinapic acid, and syringic acid are likely the main “SOS” substances used by ryegrass to resist the invasion of *Spodoptera frugiperda*. This study found that all three phenolic substances can interfere with the feeding and growth of *S. frugiperda* larvae by inhibiting their digestion and absorption, making them potential substances for developing antifeedants for *S. frugiperda*. In addition, compared with sinapic acid and syringic acid, vanillic acid not only inhibited the pupation rate of *S. frugiperda* but also had a negative impact on their longevity as adults. Notably, when the growth of *S. frugiperda* in the larval stage was restricted by sinapic acid and syringic acid, the adult longevity was prolonged. The appearance of this self-compensation phenomenon in the adult stage makes the development of its comprehensive prevention and control strategy potentially more difficult.

## 1. Introduction

Agriculture is the foundation of stable economic development and an important factor for the smooth development of other industries [1,2]. However, under natural environmental conditions, almost all crops are susceptible to pest infestations [1]. According to statistics, the global crop yield reduction caused by pest stress is close to 20% of the total agricultural production each year [1,3]. Pest stress has become one of the main limiting factors for agricultural production in China and even globally [1,3]. At the same time, some invasive pests are rapidly spreading globally, which undoubtedly exacerbates the threat of pest stress to agricultural production [4].

Unlike animals that can move, plants are immobile and can only respond to pest invasion by activating defense mechanisms [5]. There are approximately 200,000 defense-related secondary metabolites in plants [5,6,7]. Phenolic substances mainly exist in plant epidermal cells, with one or more similar phenolic structural units, and are the most abundant secondary metabolites in plants [5,6,7,8,9]. Although they do not directly participate in plant growth, they are the “executors” of plants’ chemical defenses and are therefore also known as the “SOS” substances of plants [6,10,11,12,13]. In a study on targeting Lepidoptera pests such as *Spodoptera litura* [14], *Helicoverpa armigera* [15], *Lymantria dispar* [16], and *Hyphantria cunea* [17,18,19], it was found that phenolic acids, flavonoids, and other phenolic substances have varying degrees of toxic effects on these feeding pests. Even though they decompose quickly in the environment, the residual amount needed for these toxic effects is very small. [20]. These phenolic substances therefore have the potential to create antifeedant, pesticide enhancers, or candidate substances for plant-based pesticides. This would effectively reduce the use of highly toxic chemical pesticides in the process of integrated pest management and could be used as alternative control measures for integrated pest management [20,21]. However, due to the different biological characteristics of herbivorous pests, their tolerance to different secondary metabolites often varies greatly [14,15,16,17]. For example, low concentrations of flavonoids do not have a significant effect on *H. armigera*, but they can significantly inhibit the serine protease, trypsin, and esterase activities of *S. litura* [14,15]. Therefore, in order to obtain effective active ingredients for controlling herbivorous pests, it is necessary to focus on their particular feeding relationship with their host plants.

According to the preliminary research conducted by the author’s team, it was found that after the host plant ryegrass activated a chemical defense mechanism, the content of phenolic substances such as vanillic acid, sinapic acid, and syringic acid significantly increased [2]. This led to the inhibition of mixed-function oxidases and carboxylesterase activity in the feeding *S. frugiperda* larvae [2]. Therefore, it was speculated that these phenolic substances may have a negative impact on the growth of *S. frugiperda*, which might be of great significance for the formulation of a future comprehensive control strategy for the pest. However, the chemical defense network activated by host plants is often large and the energy transfer is complex [2]. In addition to involving the metabolism of secondary metabolites such as phenols, it may also cause fluctuations in the content of some nutrients and other physical defense substances [22,23,24]. Therefore, in order to verify whether the above speculation was valid, relevant research needs to be conducted to provide direct evidence.

This study used a major agricultural invasive pest, *S. frugiperda*, as the experimental material, with vanillic acid, sinapic acid, and syringic acid as exogenous additives. The changes in growth- and digestion-related indicators were measured after feeding the larvae to assess the tolerance of *S. frugiperda* to the three phenolic substances and to determine whether they have the potential to become alternative natural products for its comprehensive control strategies.

## 2. Materials and Methods

### 2.1. Experimental Insects

The eggs and artificial diet of *S. frugiperda* were purchased from Henan Jiyuan Baiyun Industry Co., Ltd located in Henan Province, China. A 10% formaldehyde solution was used as a disinfectant for the eggs, and after soaking for 40 min, the surfaces of the eggs were rinsed with distilled water and then air dried. The eggs were incubated in an environment with a temperature of 25 °C ± 1 °C and a relative humidity of around 40%.

### 2.2. Experimental Treatment

Vanillic acid (CAS: 121-34-6), sinapic acid (CAS: 530-59-6), and syringic acid (CAS: 530-57-4) were purchased from Macklin, Source Leaf Biotechnology, and Sigma, respectively. The three phenolic substances were evenly mixed with the artificial diet in mass ratios of 1:200 and 1:40, respectively, and labeled as the vanillic acid treatment group (Va1, Va2), sinapic acid treatment group (Si1, Si2), syringic acid treatment group (Sy1, Sy2), and control group (CK).

### 2.3. Determination of Larval Food Intake and Body Weight

Referring to the method used by Zhang et al. [25], when the larvae in the treatment or control group reached the 4th, 5th, and 6th instars, newly molting larvae were selected, and the weight of the artificial diet and larvae before feeding was recorded. The artificial diet weight and body weight of the larvae were measured after 24 h, and the food intake of the 4th, 5th, and 6th instars was calculated. A total of 3 repetitions were set up, with 10 larvae in each repetition.

### 2.4. Determination of Protein and Digestive Enzyme Activity of Larvae

During the experiment, 4th-, 5th-, or 6th-instar larvae that had molted within the past 48 h were selected and stored at −80 °C to measure the protein content and digestive enzyme activity. Three replicates were set up, using physiological saline as the extraction solution. The larvae were ground into a homogenate under an ice bath and centrifuged at 10,000 rpm for 10 min at 4 °C, and the supernatant was used as the test solution. The determination of protein content followed the method of Xu et al. [26], using the Coomassie Brilliant Blue method, which uses a UV spectrophotometer to measure the absorbance changes of the supernatant mixed with Coomassie Brilliant Blue at 595 nm. The determination of pepsin activity followed the method of Zhang et al. [27]. Casein solution was added to the heated test solution, followed by further heating. Trichloroacetic acid was added and centrifuged at 3000 rpm for 5 min. Then, sodium carbonate solution and Follin-phenol reagent were added. After heating, the absorbance was measured with a spectrophotometer at 680 nm to calculate pepsin activity. The determination of α-amylase activity followed the method of Khokhar et al. [28], where the supernatant was the enzyme containing the test solution. It was incubated at room temperature for 15 min and then incubated at 40 °C for 5 min after adding dinitrosalicylic acid. A sodium potassium tartrate solution was added to terminate the reaction, and, after cooling, the absorbance was measured at 560 nm to calculate enzyme activity. The determination of trypsin activity followed the method of War et al. [29]. The supernatant was mixed with a glycine–NaOH buffer and heated. Then, the reaction was terminated by adding acetic acid. The absorbance was read at 410 nm using a UV spectrophotometer and the trypsin activity was calculated.

### 2.5. Determination of Pupa Weight, Pupation Rate, Emergence Rate, and Adult Longevity

The pupae were weighed between 12 h and 24 h after the larvae had pupated. The weight of every 10 pupae was one replicate, with three replicates in total. During the experiment, using 100 insects as a replicate, the number of *S. frugiperda* that pupated in the different treatment groups or control groups was recorded every 12 h to calculate the pupation rate. After successful metamorphosis of the adults, they were fed with honey water until they died naturally. In this process, the total number of adults was used to calculate the emergence rate, and the total longevity of the adults was also recorded.

### 2.6. Statistical Analysis

The experimental data was analyzed by SPSS 19.0 software. Duncan’s multiple comparison method using one-way ANOVA was used to test the significance of differences in various indicators between the control and treatment groups at a level of 0.05. Origin 2021 software was used for image creation.

## 3. Results

### 3.1. Changes in Larval Food Intake of S. frugiperda

**Figure 1:**
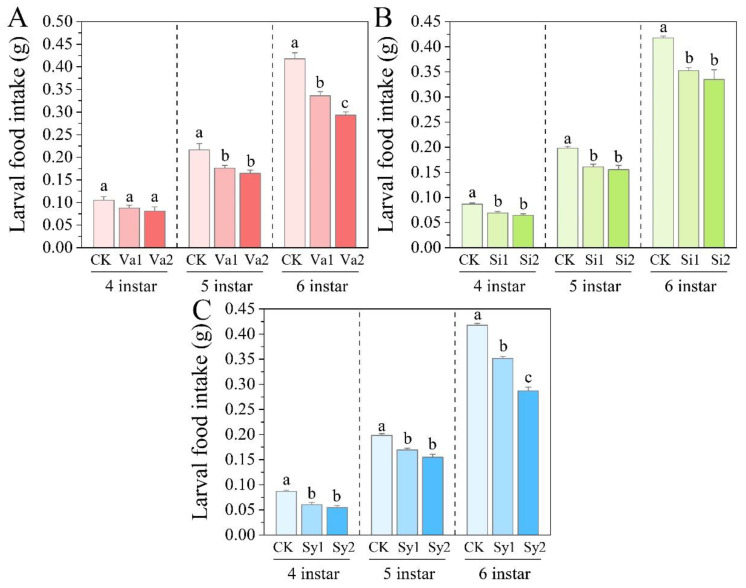
Changes in food intake of the 4th–6th-instar larvae of *Spodoptera frugiperda* after feeding on an artificial diet supplemented with vanillic acid (**A**), sinapic acid (**B**), or syringic acid (**C**), respectively. Values in the figure are mean ± standard deviation. According to Duncan’s multiple comparison, there is no significant difference between the same letters (*p* > 0.05). The same applies below.

After feeding on the experimental artificial diet supplemented with vanillic acid, sinapic acid, or syringic acid, the food intake of the 4th–6th-instar larvae of *S. frugiperda* showed similar changes, with varying degrees of reduction and concentration effects (Figure 1). Compared with the control group, the food intake of the fourth-instar larvae in the vanillic acid treatment group did not show a significant difference (*p* > 0.05), but the food intake of all other treatment groups decreased significantly (*p* < 0.05) (Figure 1A). In particular, the food intake of the sixth-instar larvae decreased by 19.59% and 29.88% in the Va1 and Va2 treatment groups, respectively, while the effects of the Si1 and Si2 treatments on their food intake were similar, decreasing by 15.62% and 19.86%, respectively, while Sy1 and Sy3 decreased by 15.83% and 31.25%, respectively (Figure 1B,C).

### 3.2. Changes in Larval Body Weight of S. frugiperda

**Figure 2:**
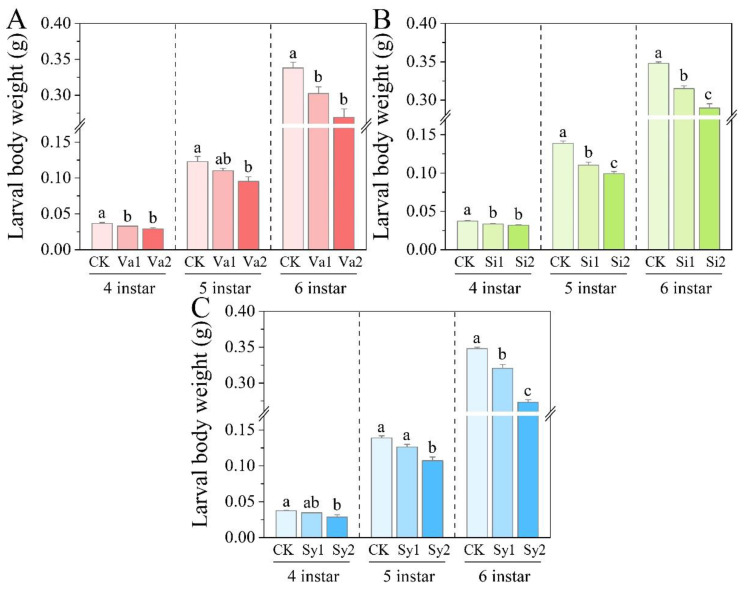
Changes in body weight of the 4th–6th-instar larvae of *S. frugiperda* after feeding on an artificial diet supplemented with vanillic acid (**A**), sinapic acid (**B**), or syringic acid (**C**), respectively.

After feeding *S. frugiperda* with the artificial diet containing the different phenolic substances, the weight gain of its 4th–6th-instar larvae was inhibited (Figure 2). The weight of the fourth- and sixth-instar larvae in the Va1 treatment group significantly decreased (*p* < 0.05), reaching 89.15% and 89.46% of the control, respectively. Although the weight of the fifth-instar larvae decreased to 89.51% of the control group weight, it did not reach a significant difference (*p* > 0.05) (Figure 2A). After feeding on the artificial diet supplemented with sinapic acid, the body weight of the 4th–6th-instar larvae of *S. frugiperda* was significantly lower than the control level (*p* < 0.05) (Figure 2B). However, in the syringic acid treatment group, compared with the control group, the inhibitory effect of low-dose Sy1 treatment on the body weight of the fourth- and fifth-instar larvae did not reach a significant difference (*p* > 0.05), and it only had a significant inhibitory effect on the growth in the weight of the sixth-instar larvae, which was 92.12% of the control group weight. However, Sy2 treatment significantly inhibited the weight growth of the 4th–6th-instar larvae (*p* < 0.05) (Figure 2C).

### 3.3. Changes in Protein Content of S. frugiperda Larvae

**Figure 3:**
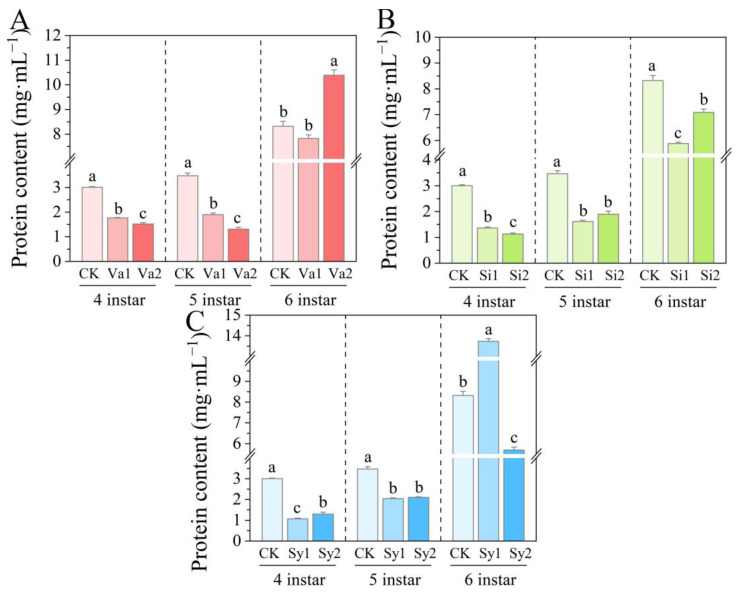
Changes in protein content of the 4th–6th-instar larvae of *S. frugiperda* after feeding on an artificial diet supplemented with vanillic acid (**A**), sinapic acid (**B**), or syringic acid (**C**), respectively.

Compared with the control group, after feeding on the artificial diet supplemented with vanillic acid, sinapic acid, or syringic acid, the protein content in the 4th–5th-instar larvae of *S. frugiperda* significantly decreased (*p* < 0.05, Figure 3). However, when the larvae developed to the sixth instar, the protein content of *S. frugiperda* in the Va2 and Sy1 treatment groups significantly increased, reaching 124.84% and 165.07% of the control, respectively. Conversely, the protein content of *S. frugiperda* fed with sinapic acid remained significantly lower than the control level, with the Si1 and Si2 treatment groups reaching 70.72% and 85.17% of the control, respectively.

### 3.4. Changes in Pepsin Activity of S. frugiperda Larvae

**Figure 4:**
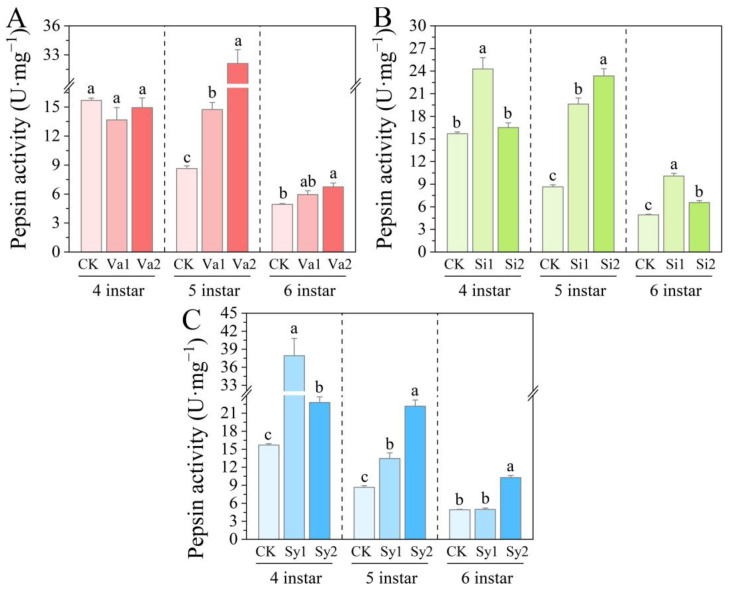
Changes in gastric protease activity in the 4th–6th-instar larvae of *S. frugiperda* after feeding on an artificial diet supplemented with vanillic acid (**A**), sinapic acid (**B**), or syringic acid (**C**), respectively.

After feeding on the artificial diet supplemented with vanillic acid, the pepsin activity of the fourth-instar larvae of *S. frugiperda* was inhibited (Figure 4A). The Va1 and Va2 treatment groups decreased to 87.07% and 95.19% of the control, respectively, but the pepsin activity in the fifth-instar larvae significantly increased (*p* < 0.05). However, after the larvae developed to the sixth instar, although the enzyme activity of the low-dose Va1 treatment group increased to 120.59% of the control, it did not reach a significant difference (*p* > 0.05), while the Va2 treatment group reached 136.73%, which is significantly higher than the control (*p* < 0.05). After feeding on the artificial diet supplemented with sinapic acid, the pepsin activity of the 4th- to 6th-instar larvae of *S. frugiperda* showed an increasing trend, especially in the 5th- to 6th-instar larvae of the Si1 and Si2 treatment groups, where enzyme activity was significantly higher than the control (*p* < 0.05) (Figure 4B). However, the pepsin activity of the 4th–5th-instar larvae fed on the artificial diet supplemented with syringic acid showed a significant increase (*p* < 0.05). But after the larvae reached the sixth instar, only the pepsin activity of the Sy2 treatment group was significantly higher than the control (*p* < 0.05), reaching 208.33% of the control. The pepsin activity of the Sy1 treatment of the sixth-instar group was close to the control (*p* > 0.05) (Figure 4C).

### 3.5. Changes in Trypsin Activity of S. frugiperda Larvae

**Figure 5:**
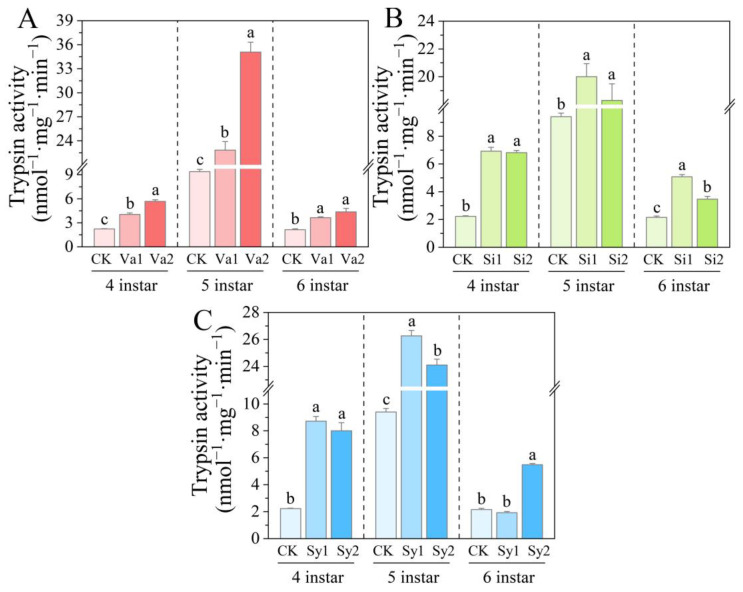
Changes in trypsin activity in the 4th–6th-instar larvae of *S. frugiperda* after feeding on an artificial diet supplemented with vanillic acid (**A**), sinapic acid (**B**), or syringic acid (**C**), respectively.

After feeding *S. frugiperda* with the three types of supplemented artificial diet, the trypsin activity of the 4th–5th-instar larvae showed a similar trend of significant increase compared to the control level (*p* < 0.05) (Figure 5). After the larvae developed to the sixth instar, though lower than the fourth and fifth instars overall, the enzyme activities of the Va1, Va2, Si1, and Si2 treatment groups still significantly increased (*p* < 0.05) when compared to the control, reaching 169.09%, 203.06%, 236.09%, and 160.95% of the control group, respectively (Figure 5A,B). There was no significant change in the Sy1 treatment group compared to the control group (*p* > 0.05), while the Sy2 treatment group was significantly higher than the control (*p* < 0.05), reaching 255.16% of the control (Figure 5C).

### 3.6. Changes in α-Amylase Activity of S. frugiperda Larvae

**Figure 6:**
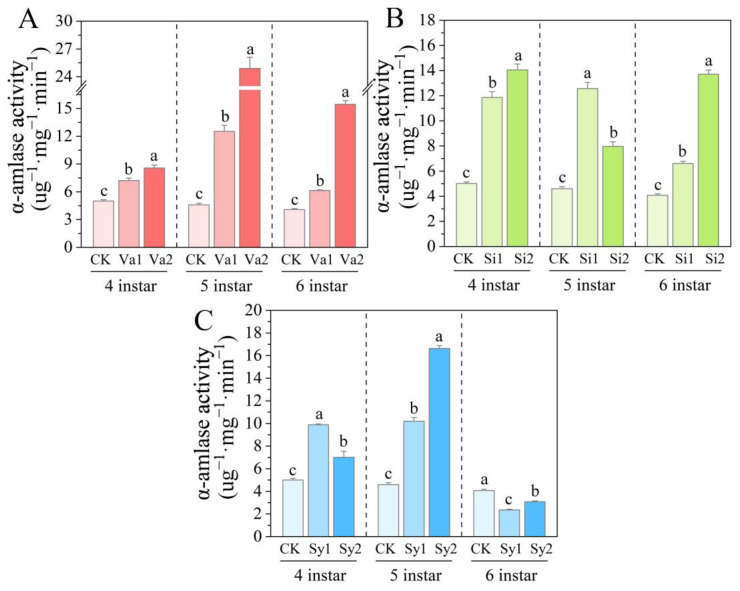
Changes in α-amylase activity in the 4th–6th-instar larvae of *S. frugiperda* after feeding on an artificial diet supplemented with vanillic acid (**A**), sinapic acid (**B**), or syringic acid (**C**), respectively.

After consuming the feed supplemented with vanillic acid, sinapic acid, or syringic acid, the trypsin activity of the 4th–5th-instar *S. frugiperda* larvae showed a similar trend of significant increase (*p* < 0.05) (Figure 6). After developing to the sixth instar, the enzyme activities of the Va1, Va2, Si1, and Si2 treatment groups still significantly increased (*p* < 0.05) (Figure 6A,B), reaching 150.55%, 379.01%, 161.94%, and 336.55% of the control, respectively. In contrast, the enzyme activities of the Sy1 and Sy2 treatment groups were significantly lower than the control levels (*p* < 0.05), reaching 57.94% and 75.44% of the control, respectively (Figure 6C).

### 3.7. Changes in Pupation Rate and Pupal Weight of S. frugiperda

**Figure 7:**
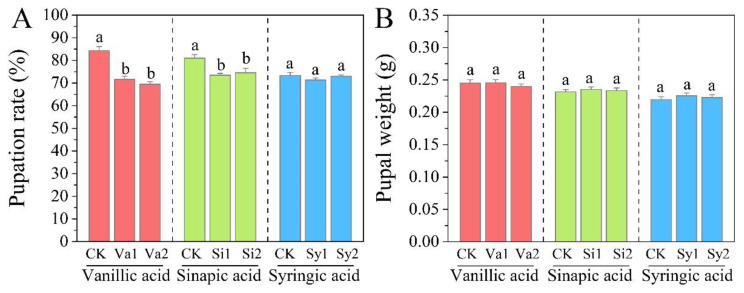
Changes in pupation rate (**A**) and pupal weight (**B**) of *S. frugiperda* larvae fed with an artificial diet supplemented with vanillic acid, sinapic acid, or syringic acid, respectively.

Compared with the control group, the pupation rate of *S. frugiperda* decreased during the larval stage after feeding on feed supplemented with vanillic acid, sinapic acid, and syringic acid (Figure 7A). The pupation rates of the Va1, Va2, Si1, and Si2 treatment groups were significantly lower than the control level (*p* < 0.05), namely 71.56%, 69.49%, 73.49%, and 74.51%, respectively. Although the magnitude of the change in pupal weight in the treatment groups varied slightly, there was no significant difference compared with the control group (*p* > 0.05) (Figure 7B).

### 3.8. Changes in the Emergence Rate and Adult Longevity of S. frugiperda

**Figure 8:**
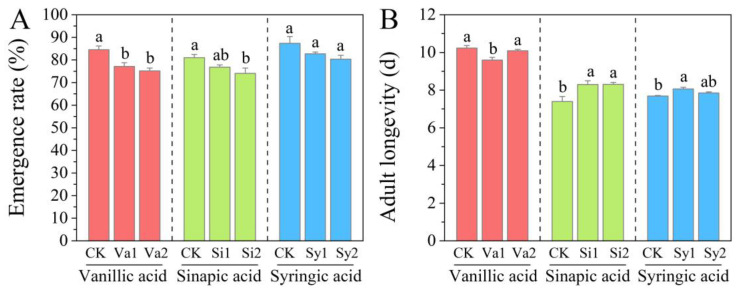
Changes in emergence rate (**A**) and adult stage (**B**) of *S. frugiperda* larvae fed with an artificial diet supplemented with vanillic acid, sinapic acid, or syringic acid, respectively.

After feeding on the artificial diet supplemented with vanillic acid, sinapic acid, or syringic acid, the larvae showed similar changes in emergence rate, all showing a decrease in emergence rate (Figure 8A). Among them, the emergence rates in the Va1, Va2, and Si2 treatment groups were significantly lower than those in the control group (*p* < 0.05), which were 77.12%, 75.16%, and 74.07%, respectively. There was no significant difference between the other treatment groups and the control group (*p* > 0.05). However, there were significant differences in the longevity of adult insects between the different treatment groups. The Va1 treatment resulted in a reduction in the adult period, while the other treatment groups showed a prolonged adult period, especially the sinapic acid treatment group. Compared with the control group, the adult longevity of Si1 and Si2 was significantly prolonged, specifically to 112.17% and 112.30% of that of the control group, respectively (*p* < 0.05) (Figure 8B).

## 4. Discussion

Previous toxicological studies have shown that some phenolic substances such as tannin, coumarin, and rutin may have the potential to become antifeedants, pesticide substitutes, or candidate substances for plant-based pesticides [14,17,30]. This study added vanillic acid, sinapic acid, or syringic acid to the artificial diet of larval *S. frugiperda* and measured the digestive enzyme activity, protein content, food intake, and weight changes of the larvae. The research scope was extended to the pupal and adult stages, further analyzing the effects on pupation rate, pupal weight, emergence rate, and adult longevity, in order to determine the tolerance of *S. frugiperda* to the three phenolic substances. The results of this study are expected to provide an important reference and guidance for improving the ecofriendly and efficient prevention and control strategies of invasive pests.

In a study on targeting Lepidoptera pests such as *H. cunea* [30], *Diaphania hyalinata* [31], *Tuta absoluta* [31], and *Plutella xylostella* [32], it was found that phenolic substances such as coumarin mainly attack their protective and detoxifying defense mechanisms. In this way, the metabolic balance of feeding pests is disrupted, thereby causing toxic damage and limiting the growth and development of the larvae [33,34]. The results of the current study indicate that phenolic substances further have a direct intervention effect on the digestive physiological processes of feeding pests. After feeding on an artificial diet supplemented with vanillic acid, sinapic acid, or syringic acid, the trypsin activity of the 4th–6th-instar larvae of *S. frugiperda* significantly increased (*p* < 0.05). This is different from the research results of Su et al. [14], as the addition of rutin, chlorogenic acid, and naringin to the feed of *S. litura* inhibited the trypsin activity of its larvae. Even though *S. frugiperda* and *S. litura* are both members of the family Noctuidae in the order Lepidoptera, their tolerance to phenolic substances may differ significantly. This is likely related to their biological characteristics, which have also been confirmed in previous studies on Lepidoptera insects [14,15,16,17]. After feeding on an artificial diet supplemented with vanillic acid, the pepsin activity in the 4th-instar larvae of *S. frugiperda* was significantly inhibited (*p* < 0.05), and the pepsin activity increased significantly with age (*p* < 0.05). This indicates that as age increases, the tolerance of *S. frugiperda* also increases. After feeding on an artificial diet supplemented with sinapic acid or syringic acid, the pepsin activity in the 4th–6th-instar larvae showed a significant increase (*p* < 0.05). Vanillic acid and sinapic acid have similar effects on α-amylase, both causing a significant increase in larval enzyme activity (*p* < 0.05). Although the α-amylase activity of the 4th–5th-instar larvae fed with an artificial diet supplemented with syringic acid also showed an increase, the enzyme activity of 6th-instar larvae was significantly inhibited (*p* < 0.05). This indicates that there are differences in the sensitivity of *S. frugiperda* to the three phenolic substances. As the larvae develop through each instar, although their tolerance increases, this tolerance still cannot mitigate the effects of the three phenolic substances on digestion through their own metabolic regulation.

In addition to digestion, the absorption process also directly affects the physiological metabolism of insects, so the two can reflect the tolerance of herbivorous insects to varying degrees [25]. Feeding is the main way for insects to obtain nutrients, and adding vanillic acid, sinapic acid, or syringic acid to an artificial diet significantly reduces the protein content of 4th–5th-instar larvae of *S. frugiperda* (*p* < 0.05). Therefore, all three phenolic substances can inhibit the absorption of nutrients by larvae. However, in the treatment group with vanillic acid (Va2) and syringic acid (Sy1), there was an increase in protein content after the larvae developed to the sixth instar, which may be related to the dosage of phenolic substances added and the increased food intake of older larvae. Since *S. frugiperda* is a type of gluttonous pest, its food intake increases with instar, especially for the last-instar larvae, whose food intake can even exceed the sum of all previous instars [35]. So, when the intake increases, the inhibitory effect of vanillic acid and syringic acid on the absorption of larvae is likely to be alleviated.

However, this relief effect is limited because digestion is inhibited. Compared with the control group, the feeding amount of *S. frugiperda* larvae in the treatment group still showed a significant decrease (*p* < 0.05). This indicates that vanillic acid, sinapic acid, and syringic acid have significant effects in inhibiting larval feeding and are potential substances for developing feeding inhibitors for *S. frugiperda*. In addition, the three phenolic substances affect their absorption and feeding by inhibiting digestion while also limiting the weight gain of the larvae. Although this has a relatively small impact on pupal weight, it seriously affects the pupation rate and further inhibits the adult emergence rate. There were also significant differences in the effects of the three phenolic substances on the longevity of the adult insects. In the vanillic acid treatment group, the adult stage was shortened, while in the sinapic acid and syringic acid treatment groups, the adult stage was extended to varying degrees. Although the adult stage is the main stage for insects to mate and lay eggs, the adult stage of *S. frugiperda* is generally 7–21 days, and its peak period for mating and laying eggs mainly occurs in the early stages [13,36,37]. Even though the treatment with sinapic acid and syringic acid extended the adult longevity of *S. frugiperda*, its effect on reproduction is unknown, and this will be the focus of further research. This effect has also sounded the “alarm” for the development of antifeedants in the future. Even though the growth of *S. frugiperda* in the larval stage was inhibited, it could “self-compensate” by prolonging the adult stage. Whether this phenomenon is a common occurrence in invasive pests or only related to the invasion mechanism of *S. frugiperda* remains to be further studied and explored.

In natural environments, insect populations often consist of overlapping generations [36]. Although antifeedants do not directly eliminate pests, substances such as vanillic acid can interfere with larval growth and digestive metabolism, potentially reducing the emergence rate of adults. This can help alleviate the pressure of subsequent control efforts. By weakening pest development at larval stages, antifeedants may enhance the effectiveness of strategies such as adult repellents or attractants and hold significant potential for future integrated pest management strategies.

## 5. Conclusions

Vanillic acid, sinapic acid, and syringic acid can all interfere with the feeding and growth of *S. frugiperda* larvae by inhibiting their digestion and absorption. All three substances have the potential to be used as antifeedants for *S. frugiperda*. However, unlike sinapic acid and syringic acid, vanillic acid not only inhibited the pupation rate of *S. frugiperda* but it also had a negative impact on their adult longevity. It is worth noting that when the growth of *S. frugiperda* in the larval stage is restricted (as it was with sinapic acid and syringic acid), it is likely to develop a “self-compensation” phenomenon in the adult stage. It is not clear whether this is directly related to its invasion mechanism, and the impact of this phenomenon on future prevention and control strategies needs to be clarified. This serves as a warning for the development of comprehensive prevention and control strategies for *S. frugiperda*. The results of this study not only provide possibilities for the ecofriendly control of *S. frugiperda* but also provide important references for the development of integrated pest control strategies in the future.

## Data Availability

The datasets generated and/or analyzed during the current study are available from the corresponding author on reasonable request.
